# Worldwide Web: High Venom Potency and Ability to Optimize Venom Usage Make the Globally Invasive Noble False Widow Spider *Steatoda nobilis* (Thorell, 1875) (Theridiidae) Highly Competitive against Native European Spiders Sharing the Same Habitats

**DOI:** 10.3390/toxins14090587

**Published:** 2022-08-26

**Authors:** Sean Rayner, Aiste Vitkauskaite, Kevin Healy, Keith Lyons, Leona McSharry, Dayle Leonard, John P. Dunbar, Michel M. Dugon

**Affiliations:** 1 Venom Systems & Proteomics Lab, School of Natural Sciences, Ryan Institute, National University of Ireland Galway, H91 TK33 Galway, Ireland; 2 Macroecology Lab, School of Natural Sciences, Ryan Institute, National University of Ireland Galway, H91 TK33 Galway, Ireland

**Keywords:** noble false widow spider, *Steatoda nobilis*, venom yield, venom potency, venom optimization, invasive species, competition, intraguild predation, predatory behaviour

## Abstract

Venom compositions include complex mixtures of toxic proteins that evolved to immobilize/dissuade organisms by disrupting biological functions. Venom production is metabolically expensive, and parsimonious use is expected, as suggested by the venom optimisation hypothesis. The decision-making capacity to regulate venom usage has never been demonstrated for the globally invasive Noble false widow *Steatoda nobilis* (Thorell, 1875) (Theridiidae). Here, we investigated variations of venom quantities available in a wild population of *S. nobilis* and prey choice depending on venom availability. To partially determine their competitiveness, we compared their attack rate success, median effective dose (ED_50_) and lethal dose (LD_50_), with four sympatric synanthropic species: the lace webbed spider *Amaurobius similis*, the giant house spider *Eratigena atrica*, the missing sector orb-weaver *Zygiella x-notata*, and the cellar spider *Pholcus phalangioides*. We show that *S. nobilis* regulates its venom usage based on availability, and its venom is up to 230-fold (0.56 mg/kg) more potent than native spiders. The high potency of *S. nobilis* venom and its ability to optimize its usage make this species highly competitive against native European spiders sharing the same habitats.

## 1. Introduction

With 50,266 species described so far [1], the order Araneae form a hyper-diverse, globally distributed clade of predatory arthropods of ecological and economic importance. Spiders are thought to consume 400–800 million tons of prey each year [2], including agricultural pests such as aphids [3] and medically important disease vectors such as mosquitos and house flies [4,5,6]. However, despite their ecological importance, little is known about intraguild competition when alien invasive spiders become established in a new area. It has been previously established that alien spiders can displace native spiders through various mechanisms. In New Zealand, displacement of the native *Latrodectus katipo* occurred after the arrival of *Steatoda capensis* from South Africa, which has a higher reproductive rate [7]. In North America, *Steatoda borealis* was displaced in some of its range after the arrival of the European *Steatoda bipunctata* [8]. In Maine, USA, the European alien spider *Linyphia triangularis* was shown to be displacing the native spider *Frontinella communis*, of which one of the mechanisms identified was web invasion [9]. Other species currently recognised for expanding their range globally include *Uloborus plumipes* [10,11], *Latrodectus geometricus* [12], *Argiope bruennichi* [13], *Cheiracanthium punctorium* [14], *Latrodectus hasselti* [15] and *Zoropsis spinimata* [16,17,18].

Over the past century, a small subset of synanthropic combfoot spiders (family Theridiidae) have benefitted from an increase in global trade and expanded their distribution range along major trading routes [19,20]. Between 1850 and 2000, at least 13 alien species of Theridiidae spiders have successfully established colonies across the European continent [21]. Increasing urbanisation and encroachment into natural habitats provides synanthropic species with further room to establish and increase their range [22]. One of the most notable recent global invasive spiders belonging to the medically significant subfamily Latrodectinae is the Noble false widow *Steatoda nobilis* (Thorell, 1875) (Theridiidae).

Throughout Europe, *Steatoda nobilis* favours synanthropic habitats. They are active all year round with peak activity between August and November. In Ireland, field observations showed that *S. nobilis* is extremely common on street furniture, signposts, traffic lights, bus shelters, boundary railings of parks, and graveyards, garden furniture, garden walls, pillars, and gates [23] regularly outnumbering native spiders. The most productive habitats are east-facing iron railings, especially the point where railings connect to brick and stone walls, and wooden fences. *S*. *nobilis* is mainly active after dark and typically preys on a wide range of arthropods [23] but has also been documented preying on a lizard in Ireland [24] and a bat in Britain [25]. In southern California, USA, *S. nobilis* has been observed preying on a milkweed bug *Lygaeus kalmii kalmii* which is noxious and normally avoided by many predators, including other *Steatoda* species [26]. *S. nobilis* has a high reproductive rate, is capable of producing several egg sacs each year, each containing on average 98 eggs [23] with the ability to still produce viable clutches at least 18 months after fertilisation [27]. 

Originally described from the Macaronesian archipelagos [28], *S. nobilis* was first documented outside of its supposed native range in Britain in 1879, but its distribution was limited to a handful of port cities until the 1990’s [29] at which point reports increased throughout the southern half of England, and from the 2000s onward, northern England, Wales and Scotland [29,30,31]. Throughout the late 1990’s and the first decade of the 21st century, *S. nobilis* expanded its range to Ireland and across continental Europe, East Asia, and the Americas [12,30,31,32,33,34,35,36,37,38]. Predictive modelling suggests that *S. nobilis* has the potential to become the world’s most invasive species of spiders [30]. Reports from Italy, the United Kingdom, and the USA (i.e., California) [30] indicate that *S. nobilis* was first observed in urban areas and spread to the surrounding countryside in subsequent years. A likely explanation is that exponential growth and higher population density eventually force an overflow of specimens into semi-natural habitats [30,31]. Population density of *S. nobilis* has not been investigated yet, but observations suggest that they dominate habitats within a few years following their arrival [23,30,31,38].

**Figure 1:**
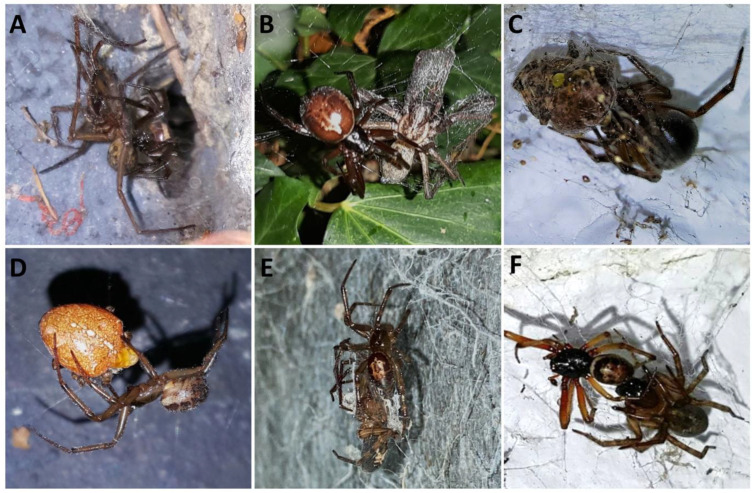
In situ predatory interactions between *Steatoda nobilis* and native European spiders. *S. nobilis* feeding on *Eratigena atrica* (**A**,**B**). *S. nobilis* feeding on European cross garden spider *Araneus diadematus* (**C**,**D**). *S. nobilis* predation on *Amaurobius similis* (**E**), and a rare occasion where *S. nobilis* has fallen prey to a native spider (*A. similis*) (**F**).

Despite several reports documenting the worldwide spread of *S. nobilis*, and concerns of ecological and human health impacts [23,24,30,31,33,39,40,41,42,43], there has been little focus on the competitiveness of *S. nobilis* against native spiders which may account for the invasiveness of *S. nobilis*. So far, no published study has quantified the invasive impact of *S. nobilis*, although previous observations in the field indicated that *S. nobilis* invades the ecological niches traditionally occupied by native synanthropic spiders and is capable of displacing them [23,42] (Figure 1). Populations of *S. nobilis* had notably increased in the urban areas of Lazio, Italy over the period of seven years, significantly reducing native spider densities in those areas [42]. Similar observations were made in Ireland, where native urban spiders were noted to be less abundant in locations colonised by *S. nobilis* [23]. Recent studies show that *S. nobilis* has a range of traits that allow it to be competitive against native species, e.g., cold tolerance, longevity up to five years, generalist diet, and high reproductive rate [23]. Additionally, *S. nobilis* shares two-thirds of its venom toxin arsenal with the “true” black widows of the genus *Latrodectus* [44], including the α-latrotoxin responsible for severe envenomation syndromes [43] and the immobilisation of vertebrate prey [24,25,45]. Both *Latrodectus* and *Steatoda* representatives have been observed feeding on small vertebrates, including mammals [25,45] and lizards [24]. During seven years of field observations, only on two occasions *S. nobilis* fell prey to native species [23]. It is likely that larger predators, such as birds, prey upon *S. nobilis* and in turn help keep numbers in check, but predation events on *S. nobilis* have yet to be documented in the literature.

On a medical point of view, symptoms from *S. nobilis* envenoming range from benign (e.g., localised pain) to severe (e.g., requiring hospitalisation), with most victims presenting a range of benign to moderate symptoms, both localised and systemic [43]. The range of symptoms and severity are likely related to several factors in both the victim, such as immune status or pathophysiological response to the venom [46,47,48,49,50], and the spider, which may be able to regulate venom delivery depending on the venom quantity available and the perceived level of threat, via venom optimization.

Venom optimization has been demonstrated in a range of venomous organisms, including snakes [49,50,51,52], centipedes [53], scorpions [54,55,56] and several spiders [47,48,57,58,59,60,61,62], and may be a process underlying all venom systems that evolved for either prey capture or a dual prey capture/defence role [63]. Venom is typically (but not exclusively) produced as a primary means to subdue prey [64] by disrupting normal physiological function, but can also aid in the pre-digestion of prey and defence against predators [44,65]. Venom production is important for survival but it is metabolically expensive to produce and is therefore a limited resource [66]. For most predators, prey capture is not guaranteed after each attempted attack [47] while in some instances, multiple prey may be acquired within a single day [59]. The “hit and miss” nature of predatory acts is such, that using all venom available in a single attack would represent an inefficient management of an important resource. This would also involve a long period of vulnerability as venom can potentially take weeks to fully replenish (e.g., 16 days for the wandering spider *Cupiennius salei* [60]). However, if the survival of the organism is at stake, using a minimum quantity of venom is risky, as failure to avoid predation may result in death [47]. Therefore, it would be advantageous for venomous organisms to modulate venom usage depending on circumstances, increasing venom quantity for defence and decreasing for predation. This “decision making” ability to regulate venom usage in the most economic context to minimise the metabolic cost associated with its use, and adapt predatory/defensive behaviour depending on venom availability to minimise risks, is known as the venom optimization hypothesis (VOH) [48,53,66]. It appears that the VOH has been mostly studied in two species of spiders, with much of this work focussed on offensive venom metering in the wandering spider *Cupiennius salei*, and a single study on defensive venom metering in the black widow spider *Latrodectus hesperus* which can also use ‘silk flicking’ or ‘attack wrap’ strategies for defence instead of depleting venom [47]. 

Despite its potential as a globally invasive species [30], its hypothesised ecological impact [23,40,41,67] and known medical importance [43], little is known about the functional ecology of *S. nobilis* or the reasons of its success establishing populations on four continents [30]. In this study, we investigated three venom-related aspects of *S. nobilis* that may contribute to its success as a global invasive. First, we established venom volume variations in wild populations of *S. nobilis* based on 550 venom extractions; second, we assessed if *S. nobilis* alter its predatory behaviour depending on venom availability; and third, we determined the competitiveness of *S. nobilis* in terms of venom potency and predation strategies against four native species of spiders found commonly in and around houses and gardens throughout Europe: The lace webbed spider *Amaurobius similis*, the giant house spider *Eratigena atrica* (both chosen because they commonly overlap habitats with *S. nobilis* and were sufficiently large to extract the amount of venom required for this study), the missing sector orb-weaver *Zygiella x-notata* (chosen because they commonly overlap habitats with *S. nobilis*), and the cellar spider *Pholcus phalangioides* (chosen as a competitor in indoor or sheltered habitats and are considered spider specialists).

Our results show a positive correlation between venom yield and body size in *S. nobilis* as previously observed for some other species [64]. We confirmed that *S. nobilis* alter its predatory behaviour based on the amount of venom available and the size of the prey it is presented with. We also found that the venom of *S. nobilis* is significantly more potent than the native European spiders we tested against each other, and that *S. nobilis* is likely a regular predator of other spiders. Overall, our findings suggest that *S. nobilis* is likely to have an impact on the urban arthropod ecosystem, as it comes to dominate the synanthropic niche occupied by a restricted number of native (e.g., *Zygiella x-notata*, *Amaurobius similis*, *Eratigena atrica*) and long established (e.g., *Pholcus phalangioides, Steatoda grossa*) species in Northern Europe.

## 2. Results

### 2.1. Spider Size and Venom Yield

We obtained venom yields and body-size measurements from a total of 550 (425 female and 125 male) *S. nobilis*, 265 (187 female and 78 male) of *Amaurobius similis*, and 139 (71 female and 68 male) of *Eratigena atrica* (Table 1). 

**Figure 2:**
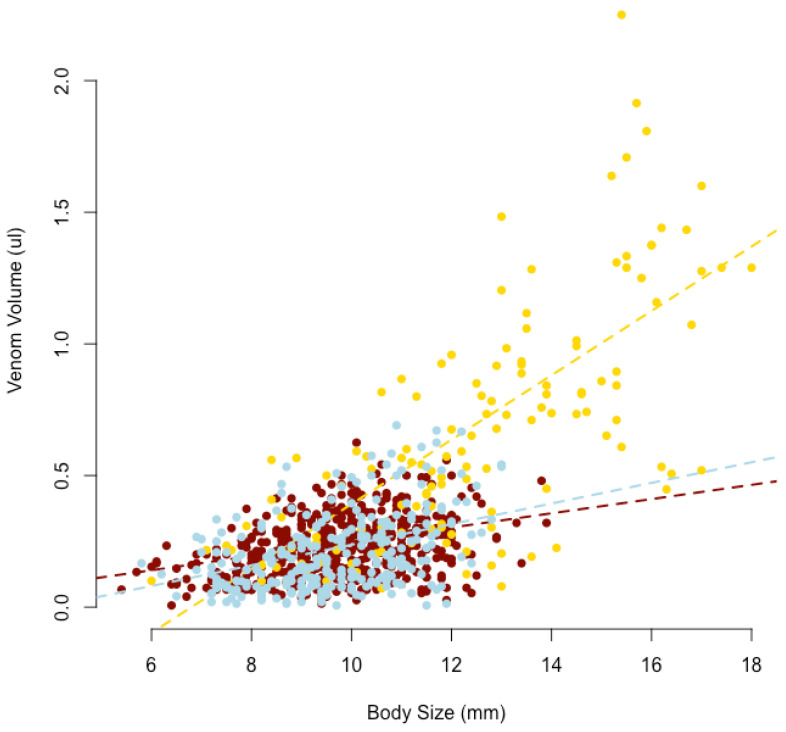
1 Relationship between venom yield and body size for *A. similis* (blue, *n* = 265), *E. atrica* (yellow, *n* = 139), and *S. nobilis* (brown, *n* = 550). Lines are fitted based on the coefficients from Table 2.

There was a significant positive relationship between body size and venom yield in *S. nobilis*, with venom yield increasing by 0.027 µL with every 1 mm increase in body size (Table 2, Figure 2). A positive relationship between venom yield and body size was also found in *A. similis* and *E. atrica*. *E. atrica* was also found to have larger increases in venom yield with increasing body size when compared to *S. nobilis* (Table 2, Figure 2) Overall, spider venom yield obtained in summer was significantly lower compared to venom yield obtained in autumn, with a decreased yield of 0.045 µL (Table 2). Sex was not found to have an effect of venom yield (Table 2).

### 2.2. Predatory Behaviour Influenced by Venom Availability

The weight range for small and large crickets was determined to be 0.019–0.048 g, and 0.352–0.633 g, respectively.

In control cohort C1 (No CO_2_ exposure nor electrostimulation), 100% (*n* = 20) of spiders attacked the large cricket in the 12 h period. In control cohort C2 (CO_2_ exposure and electrostimulation on posterior legs only) 90% (*n* = 18) of spiders attacked the prey item. In both cohorts, all attacks were successful.

Spiders in cohorts L8 and L48 were given large crickets eight and 48 h after venom extractions, respectively. In cohort L8, 50% (*n* = 10) of spiders attempted an attack, of which 35% (*n* = 7) were successful. The remaining 15% (*n* = 3) failed to subdue the prey and were observed moving away and avoiding the cricket for the remainder of the experiment. The 50% of spiders that did not attempt an attack remained in their hide, avoiding the cricket throughout the observation time, with two specimens being killed by the cricket. In cohort L48, 70% (*n* = 14) of spiders attempted an attack, of which 50% (*n* = 10) were successful. The remainder showed avoidance for the rest of the experiment. 

**Figure 3:**
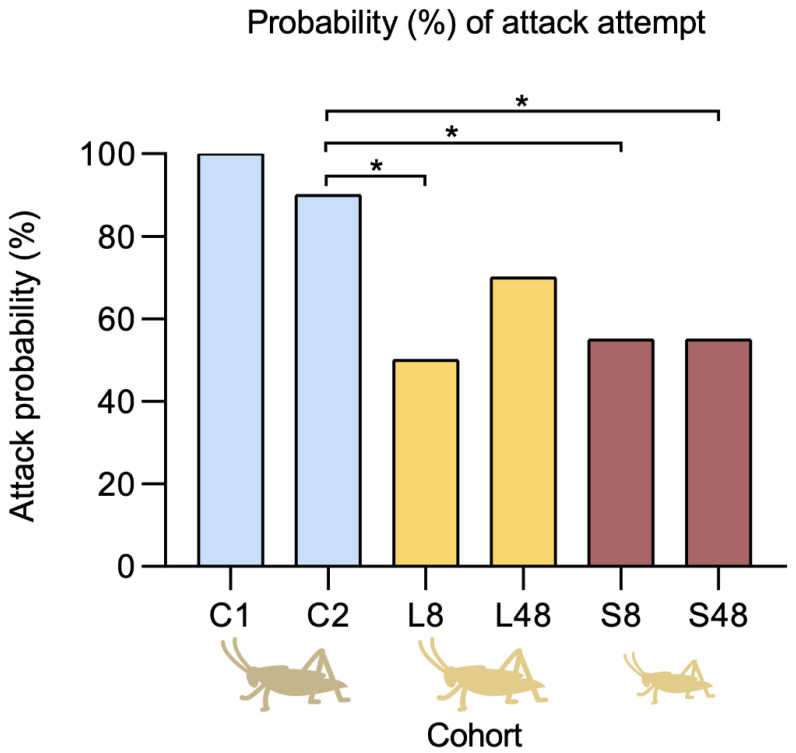
Probabilities that a spider in a respective cohort (*n* = 20) would attack a prey item. Blue bars represent the controls. Yellow bars represent spiders given large prey items 8 or 48 h after venom extraction. Brown bars represent spiders given smaller prey items 8 or 48 h after venom extraction. Cohorts significantly different compared to the control C2 from the logistic analysis (Table 3) are indicated using asterisks.

Spiders in cohorts S8 and S48 were given small crickets eight and 48 h after venom extractions, respectively. Cohort S8 had an attack rate of 55% (*n* = 11), with 50% (*n* = 10) of the attacks being successful. In total, 45% (*n* = 9) of spiders that did not attack the prey had remained hidden throughout the observations. Cohort S48 also had an attack rate of 55% (*n* = 11) with all attempts being successful. In each case the number of attacks dropped significantly when venom had been extracted, except in the case of spiders that had been given large crickets after being allowed 48 h to replenish venom yields (Figure 3). Attack probabilities for Cohort L8 (50% attack probability), Cohort S8 (55% attack probability), and Cohort S48 (55% attack probability) were significantly lower than the controls (C1: 100%; C2: 90%). While Cohort L48 had a lower attack probability (70% attack probability) it was not significantly lower compared to the controls (Table 3).

**Figure 4:**
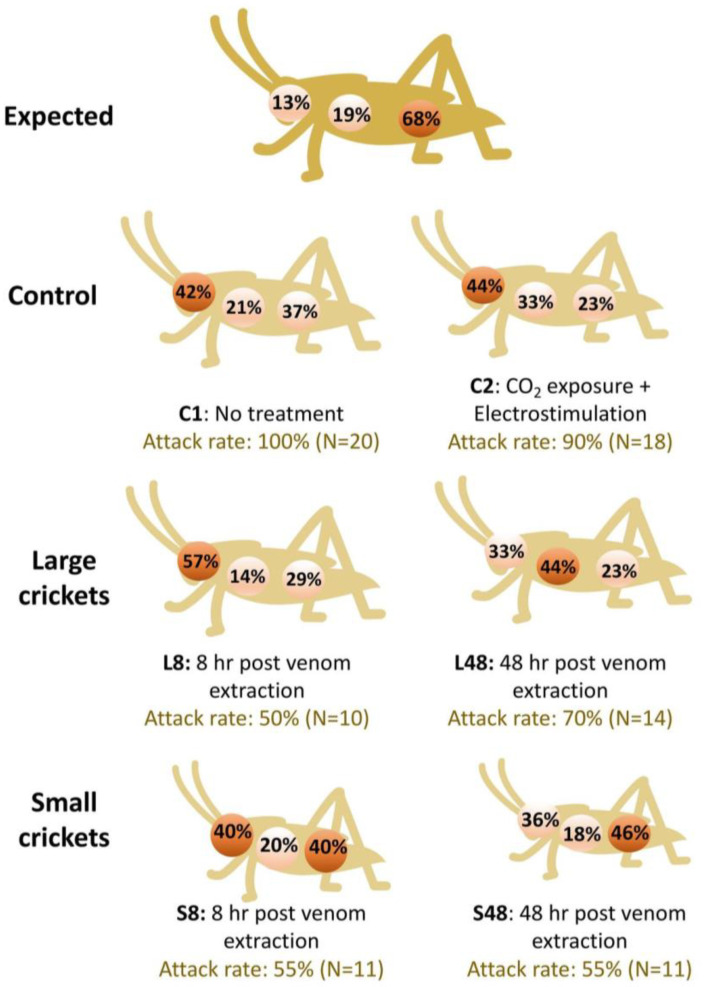
Expected and observed *Steatoda nobilis* attack rates and locations against *Gryllus assimilis* in control and test cohorts. Expected results were calculated by measuring the abdomen, thorax and head of 24 crickets and calculating the mean percentage figure that section makes up of the crickets’ entire body.

Measurements of the crickets revealed that the abdomen makes up 68% of the crickets’ overall body length, with the thorax making up 19% and the head 13%. If no preference of bite location is assumed, it would be expected that out of 20 individuals in a single cohort, 12–13 individuals would bite the abdomen, four individuals would bite the thorax and two-three individuals would bite the head of the cricket. The observed results are shown in Figure 4, with percentages representing spiders that successfully immobilised a prey item in each cohort.

### 2.3. S. nobilis, A. similis and E. atrica Venom Toxicity

30 min after being injected with 10 μL of 0.01 M PBS solution, 92% of *A. similis*, 96% of *E. atrica*, 100% of *S. nobilis* and 100% of *Z. x-notata* were alive. The impact of the PBS and the puncture wound were investigated using a Fisher’s Exact Test. Results showed that injecting spiders with PBS did not have any significant impact on the survival rate of the specimens (*p* > 0.05 in all cases).

The subset of *A. similis* (*n* = 85) that was measured had a bodyweight range of 0.050–0.179 g, a median weight of 0.08 g, and a mean weight of 0.09 g (SD = 0.030). The subset of *E. atrica* (*n* = 53) prey items had a weight range of 0.129–0.426 g, with a median weight of 0.263 g and mean weight of 0.258 g (SD = 0.074). The subsets of *S. nobilis* (*n* = 85) and *Z. x-notata* (*n* = 85) ranged in body weight between 0.091–0.281 g and 0.008–0.116 g, with median body weight values of 0.161 g and 0.060 g, and mean weight values of 0.164 g (SD = 0.044) and 0.060 g (SD = 0.024), respectively. Mean dry venom weight for 13.9 μL of pooled male and female *A. similis* venom was 1.1 mg; 6.4 mg for 42.3 μL of *E. atrica* venom, and 1.4 mg for 14.2 μL of *S. nobilis* venom.

ED_50_ and LD_50_ of *A. similis*, *E. atrica* and *S. nobilis* venoms on *A. similis*, *E. atrica*, *S. nobilis*, and *Z. x-notata* prey are outlined in Table 4. The exact LD_50_ value for *A. similis* venom on *E. atrica* prey and both ED_50_ and LD_50_ for *S. nobilis* prey could not be determined due to the test specimens not reaching the mortality of ≥50% at the highest dose tested (2%). The LD_50_ value of *E. atrica* venom on *S. nobilis* was also estimated to be above the highest dose tested (2%). Overall, *S. nobilis* venom had a notably lower ED_50_ and LD_50_ compared to the other two spider venoms (Table 4).

### 2.4. Predation Observations

**Figure 5:**
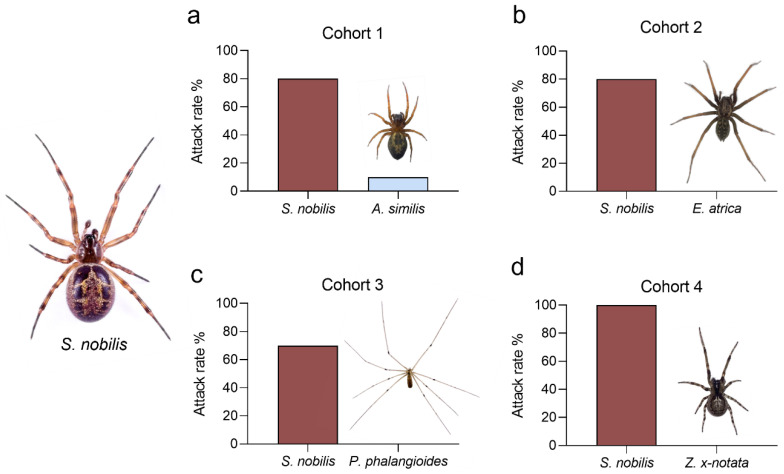
(**a**–**d**) spider attack rates in cohorts 1–4. *S. nobilis* was notably the more successful competitor in all cohorts, with only one (10%) *A. similis* successfully subduing its opponent (**a**).

In cohort 1 (*S. nobilis* and *A. similis*), 80% (*n* = 8) of *A. similis* were preyed upon by *S. nobilis* and 10% (*n* = 1) of *S. nobilis* were preyed upon by *A. similis* (Figure 5). In a span of six weeks, spiders in one enclosure (10%) did not attack one another. In cohort 2 (*S. nobilis* and *E. atrica*), 80% of *E. atrica* were preyed upon by *S. nobilis* and no *S. nobilis* fell prey to *E. atrica* (Figure 5). Spiders in one enclosure (10%) did not attack one another throughout the duration of the experiment, and one *E. atrica* (10%) in one of the merged enclosures was found dead (cause unknown). In cohort 3 (*S. nobilis* and *P. phalangioides*), 70% of *P. phalangioides* were found dead, wrapped up in *S. nobilis* silk. Spiders in the remaining three enclosures (30%) did not interact throughout the duration of the experiment. In cohort 4 (*S. nobilis* and *Z. x-notata*), all *Z. x-notata* (100%) fell prey to *S. nobilis* within the first 1–3 days after the merging of the enclosures. All *Z. x-notata* were found wrapped up in *S. nobilis* silk. 

## 3. Discussion

### 3.1. Predatory Behaviour Influenced by Venom Availability

We demonstrate that *Steatoda nobilis* optimizes its venom usage and adapts its predation behaviour based on venom availability. Irrespective of the level of venom replenishment/depletion, *S. nobilis* tended to show a preference to envenomate the cephalic region of its prey (Figure 4), where neurotoxins are likely to be more efficient [53]. In contrast, venom injected into the abdomen would likely be diluted by the circulatory system and become less effective [53] suggesting that our observed attacking behaviour may allow for a more efficient use of an energetically costly venom resource. Specimens delivered accurate bites in the head and cephalothorax only after partially wrapping the prey with silk and positioning adequately.

A significant decrease in attack rates was observed after venom extraction. The control cohort C1 (no anesthesia nor electrostimulation) had a 100% attack rate. The control cohort C2 (anesthesia and electrostimulation of the posterior legs only without venom extraction) returned a 90% attack rate, demonstrating that neither anesthesia nor electrostimulation had a significant effect on the predatory behaviour of the spiders. However, across the four test cohorts, the average attack rate falls to 55% (Figure 3). This indicates a risk assessment-like behaviour by the spider, opting for prey avoidance when venom stores are depleted or preferring to use silk over venom. Contrary to previous studies on VOH [48,53,66], prey size did not seem to affect the predatory behaviour of *S. nobilis* after venom extraction. This may be due to the relatively small size or extreme sensitivity of *Gryllus assimilis* to the venom of *S. nobilis*, which have been previously observed feeding on a range of arthropods [23] and even vertebrate prey [24,25].

### 3.2. Venom Yield and Potency

We sampled a population of *S. nobilis* and found large variations in venom yield with a significant correlation between venom yield and body size (Figure 2, Table 2). We did not find evidence of *S. nobilis* having an advantage in terms of venom volume when compared to *A. similis* and *E. atrica*. On the contrary, *E. atrica* produced significantly more venom when compared to *S. nobilis* which may partially reflect the larger maximum body size achieved by *E. atrica*. Therefore, it is unlikely that the competitive advantage of *S. nobilis* comes from larger amounts of venom injected during predatory/defensive bites. Rather, venom potency and predation/defensive strategies are more likely to play a role.

We found *S. nobilis* venom to be significantly more potent than *A. similis* and *E. atrica* venom. Previously, Wigger et al. (2002) showed that *C. salei* injected just enough venom into prey crickets *Acheta domesticus* and prey stick insects *Carausius morosus* to kill them (i.e., the amount of venom injected matched closely the LD_50_ values for these prey items). If a similar scenario is applicable to other spiders, then *S. nobilis* requires a substantially smaller proportion of its available venom to kill prey, compared to native *A. similis* and *E. atrica*, thus potentially lowering the metabolic costs associated with venom production and replenishment. Furthermore, the α-latrotoxins present in the venom allow *S. nobilis* to expand its diet to small vertebrates [24,25,44].

We found the venom of *S. nobilis* to be particularly fast-acting. A total of 93–100% of *A. similis* were immobilised within the first minute post-injection at 1–0.01% concentrations, with 100% of prey being immobilised within 2 min. At the same concentrations, 93–100% of *Z. x-notata* was immobilised within the first minute. The recent publication of the venom profile of *S. nobilis* revealed that this species shares approximately two-thirds of toxins with the closely related genus *Latrodectus* [44]. Atakuziev et al. (2014) performed comparative toxicity assays between *Steatoda* spp. (pooled *Steatoda capensis* and *Steatoda grossa* venom), *Latrodectus tredecimguttatus* and *Latrodectus hasselti* venom using African black beetles *Heteronychus arator*. The ED_100_ was significantly lower for *Steatoda* spp. (0.01 mg/kg) than *L. tredecimguttatus* or *L. hasselti* (0.06 mg/kg for both). Furthermore, the onset of paralysis occurred in a shorter time span for *Steatoda* venom than *Latrodectus* [68]. Nentwig et al. (1992) compared venom potency of the black widow spider *Latrodectus hesperus*, *Amaurobius* sp. and *Eratigena atrica* in the oriental cockroach *Blatta orientalis*. LD_50_ comparison revealed *L. hesperus* venom to be almost 7-fold more potent than *E. atrica* and 633-fold more potent than *Amaurobius sp*. venom [69]. Here, we found *A. similis* and *E. atrica* venom to be 230-fold, and 125-fold less potent than *S. nobilis* venom, respectively, when tested on *Z. x-notata* (LD_50_) (Table 4).

### 3.3. Competitiveness/Intraguild Predation

In all four cohorts, *S. nobilis* was observed to be a more successful competitor than *A. similis*, *E. atrica*, *P. phalangioides* and *Z. x-notata*. All of these spiders are web builders, utilising their webs in different ways to facilitate prey capture, as well as defence against predators [70]. At least two spider genera from the subfamily Latrodectinae, namely *Steatoda* and *Latrodectus*, construct distinct three-dimensional “tangle” webs allowing efficient prey capture [71]. Before administrating venom, *S. nobilis* employs various prey immobilisation strategies, such as prey suspension above the ground, throwing of sticky silk threads and prey wrapping [44,72]. The closely related *L. hesperus* tends to flick silk as a primary defence strategy under medium threat [47]. We have shown that *S. nobilis* utilises silk flicking and prey wrapping prior to envenomation as a means of venom conservation. Among the synanthropic species investigated here, this behaviour is specific to *S*. *nobilis* and contrast with the hunting strategies of the species it is mainly in competition with.

In addition to the VOH, which may be the norm among most spiders, these strategies allow *S. nobilis* to minimise venom expenditure and associated metabolic costs, suggesting a potential competitive advantage against native spider species. Further studies are needed to quantify the rate and extent of *S. nobilis* population establishment and assess to what extend the encroachment of *S. nobilis* affects native species. This is especially important in vulnerable biomes, such as Holarctic and Austral insular ecosystems (e.g., Ireland, Great Britain, New Zealand, Tasmania) where species richness and competition are limited. Overall, the combination of all the factors studied in this paper make the invasive potential of *S. nobilis* a cause for concern and this species should be monitored closely throughout its range.

## 4. Materials and Methods

### 4.1. Spider Collection, Housing, Measurements, and Venom Extraction

Specimens of *A. similis, E. atrica, S. nobilis, P. phalangiodes*, and *Z. x-notata* were collected from street furniture, garden walls, park railings and building windows in several locations across three Irish counties: Galway, Longford, and Dublin, between May 2018, and November 2021 (Supplementary Table S1). Sex of the specimens was determined based on the presence of the epigyne in females and swollen pedipalps in males. Each species was identified based on characteristic abdominal markings and body sizes [23,73,74,75]. All specimens were kept in 50 mL falcon tubes and transported to the lab where they were housed as per [23]. Specimens of *S. nobilis*, *E. atrica* and *A. similis* were used in the venom toxicity assay. *Z. x-notata* was also used in the assay as a test subject. All four species were also used for the predation observations experiment, with the addition of *P. phalangioides* as this species specialises in preying on spiders.

Venom extractions were carried out on *S. nobilis, A. similis* and *E. atrica* specimens using electrostimulation as per [44], within 24 h of capture. Previous studies on other spider species [76,77,78] demonstrate that factors such as sex and body size can influence the quantity of venom available. Therefore, the body sizes (prosoma + opisthosoma) of *S. nobilis, A. similis* and *E. atrica* specimens were measured using an analogue Vernier calliper. Individual venom yields, the specimen’s sex, and the months of capture/extraction were recorded to determine the relationship between the size, sex, season and venom yield.

### 4.2. Venom Toxicity Assays

*Amaurobius similis* and *E. atrica* were chosen to assess the comparative toxicity of *S. nobilis* venom. These species were selected due to their synanthropic lifestyle and overlapping habitats with *S. nobilis*, and their relatively large size, more adequate for venom extractions. Venom efficacy was assessed against four species of synanthropic spiders: *A. similis*, *E. atrica*, *Z. x-notata*, and *S. nobilis*. These species were selected in order to observe (1) the efficacy of *S. nobilis* venom on native spiders it is likely to encounter in the habitats it colonises; (2) the sensitivity of *S. nobilis* to the venom of native spiders. 

**Figure 6:**
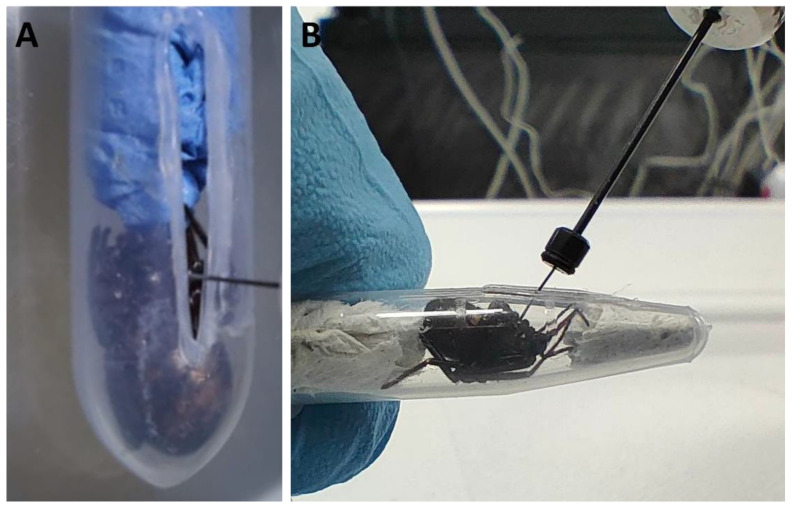
Modified Eppendorf used for restraining live spiders. (**A**) A slit was made using a soldering iron along the side of the Eppendorf. (**B**) A spider positioned at the bottom of the Eppendorf with piece of tissue placed in the remaining area to ensure that the spider was immobilised.

Control cohorts of adult *A. similis*, *E. atrica*, *S. nobilis*, and *Z. x-notata* (*n* = 25 per cohort) were injected with 10 μL of 0.01 M Phosphate Buffer Saline (PBS) solution to determine that (1) the excipient (PBS) was not toxic to the spiders and (2) that the puncture wound of the injection was not causing the impairment of the test specimens. Each live specimen was restrained with a modified Eppendorf tube (Figure 6) to prevent movement while injecting into the soft, lateral arthrodial membrane of the cephalothorax (between leg 2 and leg 3) with a 50 μL microsyringe (Hamilton Neuros syringe, model 1705RN, point style 3). The specimens were monitored 1 min, 2 min, 5 min, 10 min, 20 min, and 30 min post-injection. Death and survival at each time point were recorded.

Three to five increasing venom concentrations (0.0001–2% of “raw” venom in PBS) were administered to four species of prey: *A. similis*, *E. atrica*, *S. nobilis*, and *Z. x-notata* (*n* = 15 per group) to establish dose-related responses and to determine the median effective dose (ED_50_) and the median lethal dose (LD_50_) (Table 5). Venom solutions were prepared by performing a series of dilutions with crude *A. similis*, *E. atrica*, or *S. nobilis* venom and the PBS. Injections were performed as per control injections. The condition of each specimen was evaluated and recorded at each time point. Specimens showing no change in their normal locomotive function post-injection were marked as unaffected; spiders displaying signs of abnormal behaviour, such as reduced mobility, disorientation, were recorded as immobilised; and unresponsive specimens displaying the typical death curl position were marked as dead. ED_50_ was determined at 2 min and LD_50_ at 30 min time points post-injection. The body weights of a subset of each prey species were recorded. *A. similis*, *E. atrica*, and *S. nobilis* crude venom volume at each concentration were converted to corresponding dry venom weight per kilogram of prey bodyweight to facilitate ED_50_ and LD_50_ extrapolation.

### 4.3. Influence of Venom Availability on Behaviour

To investigate whether *S. nobilis* alter their behaviour based on the amount of venom available, we measured spider response times to prey vs. available venom; determined the location of the initial bite/envenomation (i.e., head/thorax/abdomen), and measured response type (i.e., attack or avoidance) in relation to prey size.

Female *S. nobilis* specimens were individually housed in transparent enclosures (15 × 7.5 × 6 cm) with a small hide secured in place on the right side of each enclosure. Two small doorways were cut into the lid so that prey items could be supplied with minimum disturbance. Once housed, the spiders were left undisturbed for a full week. The spiders were divided into six cohorts (*n* = 20 per cohort), based on venom extractions and prey size (Table 6). Cohorts C1 and C2 served as controls and the other four (L8, L48, S8, S48) had their venom extracted. The commercially available silent field cricket *Gryllus assimilis* was chosen as prey item, as crickets are regularly used as a prey model [53,59].

The length of the head, thorax, and abdomens of 24 crickets were measured and compared to their overall body length. A ratio was derived to predict the number of envenomations expected to that region in case of random bite (Figure 4). Cricket weight was standardised by measuring a sample of 24 specimens from each small and large cohorts. The acceptable range of weight was arbitrarily set as one standard deviation from the mean weight. Feeding was carried out at dusk, when *S. nobilis* is naturally most active, ensuring that the lights in the lab were dimmed. A cricket was dropped into each enclosure through one of the doors cut into the lid. Spider behaviour was recorded for two hours following the introduction of the prey cricket, and once again 12 h after introduction. The following variables were recorded: (1) attempts to catch/kill the cricket, (2) spider avoidance of the cricket, (3) response time of an attempted attack, (4) success rate of the attempted attack, (5) the location of the initial bite, and (6) number of cricket deaths within the 12 h period.

### 4.4. Predation Observations

We investigated if *S. nobilis* has a competitive advantage when in close proximity to native synanthropic spiders and vice versa. The aim was to determine if *S. nobilis* offensive and avoidance/defensive strategies (web construction and prey capture/wrapping techniques) give *S. nobilis* a competitive advantage over native spiders. The four synanthropic species we chose for this experiment were *A. similis*, *E. atrica*, *Z. x-notata*, and *P. phalangioides*. The latter is known to regularly catch and feed on other spiders, and as such, this species might possibly be a competitor/predator of *S. nobilis* in southern Europe and warmer parts of the world [79]. However, in Ireland, the UK, and northern Europe, *P. phalangioides* is restricted to very sheltered areas in synanthropic habitats. In Ireland (and the UK), our observations tend to show that both species rarely overlap. 

Four cohorts were established to observe predatory/competitive behaviour between *S. nobilis* and native spiders. The first cohort consisted of *S. nobilis* (*n* = 10) and *A. similis* (*n* = 10), the second—*S. nobilis* (*n* = 10) and *E. atrica* (*n* = 10), the third—*S. nobilis* (*n* = 10) and *P. phalangioides* (*n* = 10) and the fourth—*S. nobilis* (*n* = 10) and *Z. x-notata* (*n* = 10) specimens. All spiders were starved for two weeks prior to the experiment. Each spider was housed individually in a 500 mL cylindrical see-through plastic enclosure. The housing ensured sufficient airflow via multiple airholes at the side of each enclosure, and a hide was mounted at the bottom to provide cover. All enclosures were kept under standard laboratory conditions (12 h dark/light cycle, 20 °C). Each spider was kept in an individual enclosure for 3–4 days to allow it to acclimatize and to build a web. Thenceforth, two enclosures, one containing *S. nobilis* and the other one containing either *A. similis*, *E. atrica*, *P. phalangioides*, or *Z. x-notata* were merged and secured at the opening, allowing the two specimens to interact. The merged enclosures were monitored daily and the results of predatory interactions or lack thereof were recorded. Interactions were considered as predation events if the spider was observed attacking/feeding on the other spider, and if one of the spiders was found wrapped up in another spider’s silk.

### 4.5. Statistical Analysis

To test the relationship between spider venom yield, body size, sex, and seasonality we ran general linear models in R Version 1.4.1717 (Boston, MA, USA) [80] with venom yield as the response variable and body size, sex, and seasonality as explanatory variables. We included interactions between body size and both seasonality (Spring, Summer, Autumn) and sex (Female, Male) and selected the best model based on Akaike information criteria which penalizes extra effective parameters to avoid overparameterized models, to select the minimum adequate model [81]. Spider venom ED_50_ and LD_50_ were estimated using the Probit models from the Mass package in R [82]. 

For the venom optimisation experiments a logistic regression model using the generalised linear model function with binomial errors from the Mass package in R, [82] were used in order to compare the probability of an individual attack across different cohorts (Table 6). The probability of attack for each cohort was compared to the baseline control cohort 2 (C2) in the analysis. This was to determine if the behaviour of spiders differs significantly after venom extraction.

## Figures and Tables

**Table 1:** Body size (prosoma + opisthosoma) and venom yield of female and male *Steatoda nobilis*, *Amaurobius similis* and *Eratigena atrica* spiders (data shown as min-max, mean ± SD).

Species	Sex	Body Size (mm)	Venom Yield (µL)
***S. nobilis***	Female	5.70–13.90	0.01–0.63
	*n* = 425	9.85 ± 1.41	0.23 ± 0.11
	Male	5.40–12.50	0.03–0.50
	*n* = 125	9.15 ±1.23	0.19 ± 0.10
***A. similis***	Female	6.20–13.00	0.01–0.69
	*n* = 187	10.02 ± 1.5	0.24 ± 0.16
	Male	5.80–12.00	0.01–0.35
	*n* = 78	8.69 ± 1.07	0.12 ± 0.08
***E. atrica***	Female	6.00–18.00	0.03–1.71
	*n* = 71	11.98 ± 2.72	0.52 ± 0.40
	Male	7.10–17.40	0.10–2.25
	*n* = 68	12.63 ± 2.50	0.75 ± 0.48

**Table 2:** Coefficients for the model testing the relationship between *S. nobilis* (*n* = 550), *A. similis* (*n* = 265), and *E. atrica* (*n* = 139) venom yield, body size, sex (female, male), and season (autumn, spring, summer).

Fixed Factors	Estimate	Standard Error	*p*-Value
Intercept (Baseline: *S nobilis, Autumn, Female*)	−0.022	0.049	0.65
Body size (mm) (*S. nobilis*)	0.027	0.005	<0.001
Season _Spring_	−0.029	0.019	0.18
Season _Summer_	−0.045	0.012	<0.005
Sex _Male_	−0.006	0.012	0.64
Species Intercept (*E atrica*)	−0.809	0.082	<0.001
Species Intercept (*A similis*)	−0.132	0.081	0.10
*Interaction Size: E. atrica*	0.095	0.007	<0.001
*Interaction Size: A. similis*	0.012	0.008	0.145

**Table 3:** Model output from the logistic analysis of probability of attack over the course of the experiment. The estimates are given in log odds, with the intercept being the log-odds for C2. All other estimates show the difference in log-odds compared to C2. Note that a Standard Error cannot be estimated for C1 as this cohort has a 100% attack rate.

	Estimate	Standard Error	*p*-Value
Intercept (C2)	2.198	0.745	<0.05
C1	16.369	-	-
L8	−2.197	0.869	<0.05
L48	−1.350	0.891	0.130
S8	−2.00	0.87	<0.05
S48	−2.00	0.87	<0.05

**Table 4:** *Amaurobius similis*, *Eratigena atrica* and *Steatoda nobilis* venom ED_50_ and LD_50_ values when tested on *Amaurobius similis*, *Eratigena atrica*, *Steatoda nobilis*, and *Zygiella x-notata* prey (*n* = 15 per group). ED_50_ was determined at 2 min and LD_50_ at 30 min post venom administration. The values are denoted as dry protein weight (mg) per kg bodyweight of prey. SE—standard error.

	*A. similis* Venom		*E. atrica* Venom		*S. nobilis* Venom	
	**ED_50_, LD_50_** **(mg/kg)**	**SE**	**ED_50_, LD_50_** **(mg/kg)**	**SE**	**ED_50_, LD_50_** **(mg/kg)**	**SE**
**Prey**						
***A. similis***	N/A	-	ED_50_ = 10.07 LD_50_ = 445.30	0.26 0.50	ED_50_ = 0.25 LD_50_ = 0.96	0.14 90.20
***E. atrica***	ED_50_ = 34.40 LD_50_ > 60.44	0.17 -	N/A	-	ED_50_ = 0.71 LD_50_ = 0.46	0.17 0.23
***S. nobilis***	ED_50_ > 98.73 LD_50_ > 98.73	- -	ED_50_ = 4.14 LD_50_ > 188.17	0.53 -	N/A	-
***Z. x-notata***	ED_50_ = 0.59 LD_50_ = 128.50	1.46 0.27	ED_50_ = 2.39 LD_50_ = 69.80	0.31 0.16	ED_50_ = 0.09 LD_50_ = 0.56	0.22 0.14

**Table 5:** *Amaurobius similis*, *Eratigena atrica*, and *Steatoda nobilis* venom concentrations used in the toxicity assays based on the prey species. For each species the venom was pooled and homogenized. Venom concentrations are given as percentage crude spider venom diluted in 0.01 M Phosphate Buffer Saline solution. N/A = non-applicable.

Prey	Venom Concentrations (%)		
	*A. similis* Venom	*E. atrica* Venom	*S. nobilis* Venom
***A. similis***	N/A	0.01, 0.1, 1, 2	0.0001, 0.001, 0.01, 0.1, 1
***E. atrica***	0.01, 0.1, 1, 2	N/A	0.01, 0.1, 1
***S. nobilis***	0.01, 0.1, 1, 2	0.01, 0.1, 1, 2	N/A
***Z. x-notata***	0.01, 0.1, 1, 2	0.001, 0.01, 0.1, 1	0.0001, 0.001, 0.01, 0.1, 1

**Table 6:** *Steatoda nobilis* cohorts used in the predatory behaviour influenced by venom availability experiment (*n* = 20 per cohort). Cohorts C1 and C2 were control cohorts, with C1 spiders not being subjected to anaesthesia or venom extraction. To check that electrostimulation and CO_2_ exposure only (both required for venom extraction) do not negatively affect predatory response in the test cohorts, C2 spiders underwent anaesthesia and electrostimulation (on posterior legs only) without venom extraction and were offered a large cricket 8 h post electrostimulation. The remaining cohorts were both anaesthetised, had their venom extracted, and were offered either a large (L-cohorts) or small (S-cohorts) cricket 8 h or 48 h post venom extraction.

Cohort	Anaesthetised	Venom Extracted	Time (h)	Prey Size
C1	No	No	N/A	Large
C2	Yes	Electrostimulation only	8	Large
L8	Yes	Yes	8	Large
L48	Yes	Yes	48	Large
S8	Yes	Yes	8	Small
S48	Yes	Yes	48	Small

## Data Availability

Not applicable.

## Supplementary Materials

The following supporting information can be downloaded at: https://www.mdpi.com/article/10.3390/toxins14090587/s1, Table S1, Details of the collection of specimens for each species of spiders used in the experiments.

## References

[1] World Spider Catalogue (2022). World Spider Catalogue Version 23.5. Natural History Museum Bern. http://wsc.nmbe.ch.

[2] Nyffeler M., Birkhofer K. (2017). An estimated 400–800 million tons of prey are annually killed by the global spider community. Sci. Nat..

[3] Badenhausser I., Gross N., Mornet V., Roncoroni M., Saintilan A., Rusch A. (2019). Increasing amount and quality of green infrastructures at different scales promotes biological control in agricultural landscapes. Agric. Ecosyst. Environ..

[4] Barin A., Arabkhazaeli F., Rahbari S., Madani S.A. (2010). The housefly, *Musca domestica*, as a possible mechanical vector of Newcastle disease virus in the laboratory and field. Med. Vet. Entomol..

[5] Ndava J., Llera S.D., Manyanga P. (2018). The future of mosquito control: The role of spiders as biological control agents: A review. Int. J. Mosq. Res..

[6] Wang Y.-C., Chang Y.-C., Chuang H.-L., Chiu C.-C., Yeh K.-S., Chang C.-C., Hsuan S.-L., Lin W.-H., Chen T.-H. (2011). Transmission of Salmonella between Swine Farms by the Housefly (*Musca domestica*). J. Food Prot..

[7] Hann S. (1990). Evidence for the displacement of an endemic New Zealand spider, *Latrodectus katipo* Powell by the South African species *Steatoda capensis* Hann (Araneae: Theridiidae). N. Z. J. Zool..

[8] Nyffeler M., Dondale C.D., Redner J.H. (1986). Evidence for displacement of a North American spider, *Steatoda borealis* (Hentz), by the European species *S. bipunctata* (Linnaeus) (Araneae: Theridiidae). Can. J. Zool..

[9] Bednarski J., Ginsberg H., Jakob E.M. (2010). Competitive interactions between a native spider (*Frontinella communis*, Araneae: Linyphiidae) and an invasive spider (*Linyphia triangularis*, Araneae: Linyphiidae). Biol. Invasions.

[10] Rozwałka R., Rutkowski T., Bielak-Bielecki P. (2017). New data on introduced and rare synanthropic spider species (Arachnida: Araneae) in Poland (II). Ann. Univ. Mariae Curie-Sklodowska Sect. C–Biol..

[11] Suvák M. (2013). Invasive spider *Uloborus plumipes* Lucas, 1846 (*Araneae: Uloboridae*), new to Slovakia. Folia Faun. Slovaca.

[12] Taucare-Ríos A., Mardones D., Zúñiga-Reinoso A. (2016). *Steatoda nobilis* (Araneae: Theridiidae) in South America: A new alien species for Chile. Can. Entomol..

[13] Kumschick S., Fronzek S., Entling M.H., Nentwig W. (2011). Rapid spread of the wasp spider *Argiope bruennichi* across Europe: A consequence of climate change?. Clim. Change.

[14] Krehenwinkel H., Rödder D., Năpăruş-Aljančič M., Kuntner M. (2016). Rapid genetic and ecological differentiation during the northern range expansion of the venomous yellow sac spider *Cheiracanthium punctorium* in Europe. Evol. Appl..

[15] Vink C.J., Derraik J., Phillips C.B., Sirvid P.J. (2011). The invasive Australian redback spider, *Latrodectus hasseltii* Thorell 1870 (Araneae: Theridiidae): Current and potential distributions, and likely impacts. Biol. Invasions.

[16] Harvey P. (2012). *Zoropsis spinimana* (Dufour, 1820) established indoors in Britain. Newsl. Br. Arachnol. Soc..

[17] Nadolny A.A. (2016). The first record of *Zoropsis spinimana* (Aranei, Zoropsidae) in the Crimea. Zool. Ecol..

[18] Purgat P., Ondrejková N., Krumpálová Z., Gajdoš P., Hurajtová N. (2021). *Tegenaria hasperi* Chyzer, 1897 and *Zoropsis spinimana* (Dufour, 1820), newly recorded synanthropic spiders from Slovakia (Araneae, Agelenidae, Zoropsidae). Check List.

[19] Hulme P.E. (2009). Trade, transport and trouble: Managing invasive species pathways in an era of globalization. J. Appl. Ecol..

[20] Mooney H.A., Hobbs R.J. (2000). Global change and invasive species: Where do we go from here. Invasive Species in a Changing World.

[21] Kobelt M., Nentwig W. (2007). Alien spider introductions to Europe supported by global trade. Divers. Distrib..

[22] Nentwig W., Pantini P., Vetter R.S. (2017). Distribution and medical aspects of *Loxosceles rufescens*, one of the most invasive spiders of the world (*Araneae: Sicariidae*). Toxicon.

[23] Dugon M.M., Dunbar J.P., Afoullouss S., Schulte J., McEvoy A., English M.J., Hogan R., Ennis C., Sulpice R. (2017). Occurrence, reproductive rate and identification of the non-native Noble false widow spider *Steatoda nobilis* (Thorell, 1875) in Ireland. Boil. Environ. Proc. R. Ir. Acad..

[24] Dunbar J., Ennis C., Gandola R., Dugon M. (2018). Biting off more than one can chew: First record of the non-native Noble false widow spider *Steatoda nobilis* (Thorell, 1875) feeding on the native Viviparous lizard *Zootoca vivipara*. Biol. Environ. Proc. R. Ir. Acad..

[25] Dunbar J.P., Vitkauskaite A., Lawton C., Waddams B., Dugon M.M. (2022). Webslinger versus dark knight: First record of a false widow spider *Steatoda nobilis* preying on a pipistrelle bat in Britain. Ecosphere.

[26] Faúndez E., Johnson E., Angelone E.V. (2020). A case of predation by the noble false widow *Steatoda nobilis* (Thorell, 1875) (*Araneae: Theridiidae*) on the small milkweed bug *Lygaeus kalmii kalmii* Stal, 1874 (*Heteroptera: Lygaeidae*). Revista Ibérica de Aracnología.

[27] Locket G.H. (1979). Some notes on the life history of *Steatoda nobilis* (Thorell). Newsl. Br. Arachnol. Soc..

[28] Thorell T. (1875). Diagnoses aranearum Europaearum aliquot novarum. Tijdschrift voor Entomologie.

[29] Snazell R., Jones D. (1993). The theridiid spider *Steatoda nobilis* (Thorell, 1875) in Britain. Bull. Br. Arachnol. Soc..

[30] Bauer T., Feldmeier S., Krehenwinkel H., Wieczorrek C., Reiser N., Breitling R. (2019). *Steatoda nobilis*, a false widow on the rise: A synthesis of past and current distribution trends. NeoBiota.

[31] Hambler C. (2019). The Noble false widow spider *Steatoda nobilis* is an emerging public health and ecological threat. OSF Prepr..

[32] Dunbar J.P., Schulte J., Lyons K., Fort A., Dugon M.M. (2018). New Irish record for *Steatoda triangulosa* (Walckenaer, 1802), and new county records for *Steatoda nobilis* (Thorell, 1875), *Steatoda bipunctata* (Linnaeus, 1758) and *Steatoda grossa* (CL Koch, 1838). Ir. Nat. J..

[33] Faúndez E.I., Téllez F. (2016). Primer registro de una mordedura de *Steatoda nobilis* (Thorell, 1875) (*Arachnida: Araneae: Theridiidae*) en Chile. Arquivos Entomolóxicos.

[34] Vetter R.S., Adams R.J., Berrian J.E., Vincent L. (2015). The European spider *Steatoda nobilis* (Thorell, 1875) (*Araneae: Theridiidae*) becoming widespread in California. Pan-Pac. Entomol..

[35] Vetter R.S., Rust M.K. (2012). A large European combfoot spider, *Steatoda nobilis* (Thorell 1875) (*Araneae: Theridiidae*), newly established in Ventura County, California. Pan-Pac. Entomol..

[36] Abdel-Rahman M.A., Omran M.A.A., Abdel-Nabi I.M., Ueda H., McVean A. (2009). Intraspecific variation in the Egyptian scorpion *Scorpio maurus palmatus* venom collected from different biotopes. Toxicon.

[37] Zamani A., Mirshamsi O., Jannesar B., Marusik Y.M., Esyunin S.L. (2017). New data on spider fauna of Iran (Arachnida: Araneae), Part II. Zool. Ecol..

[38] Nolan M. (1999). Three Spiders (Araneae) New to Ireland: *Bolyphantes alticeps*, *Oonops domesticus* and *Steatoda nobilis*. Ir. Nat. J..

[39] Dunbar J.P., Khan N.A., Abberton C.L., Brosnan P., Murphy J., Afoullouss S., O’Flaherty V., Dugon M.M., Boyd A. (2020). Synanthropic spiders, including the global invasive noble false widow *Steatoda nobilis*, are reservoirs for medically important and antibiotic resistant bacteria. Sci. Rep..

[40] Faúndez E.I., Carvajal M.A., Aravena-Correa N.P. (2020). On a bite by *Steatoda nobilis* (Thorell, 1875) (*Araneae: Theridiidae*) on a human being, with comments on its handling during the 2020 SARS-COV-2 pandemic. Rev. Iber. Aracnol..

[41] Warrell D., Shaheen J., Hillyard P., Jones D. (1991). Neurotoxic envenoming by an immigrant spider (*Steatoda nobilis*) in southern England. Toxicon.

[42] Kulczycki A., Simeon E., Legittimo C.M., Di Pompeo P. (2012). New Records of *Steatoda nobilis* (Thorell, 1875) (Araneae, Theridiidae), an Introduced Species on the Italian Mainland and in Sardinia. Arachnology.

[43] Dunbar J.P., Vitkauskaite A., O’Keeffe D.T., Fort A., Sulpice R., Dugon M.M. (2021). Bites by the noble false widow spider *Steatoda nobilis* can induce *Latrodectus*-like symptoms and vector-borne bacterial infections with implications for public health: A case series. Clin. Toxicol..

[44] Dunbar J.P., Fort A., Redureau D., Sulpice R., Dugon M.M., Quinton L. (2020). Venomics Approach Reveals a High Proportion of *Lactrodectus*-Like Toxins in the Venom of the Noble False Widow Spider *Steatoda nobilis*. Toxins.

[45] Vitkauskaite A., Dunbar J.P., Lawton C., Dalagiorgos P., Allen M.M., Dugon M.M. (2021). Vertebrate prey capture by *Latrodectus mactans* (Walckenaer, 1805) and *Steatoda triangulosa* (Walckenaer, 1802) (Araneae, Theridiidae) provide further insights into the immobilization and hoisting mechanisms of large prey. Food Webs.

[46] Dunbar J.P., Sulpice R., Dugon M.M. (2019). The kiss of (cell) death: Can venom-induced immune response contribute to dermal necrosis following arthropod envenomations?. Clin. Toxicol..

[47] Nelsen D.R., Kelln W., Hayes W.K. (2014). Poke but don’t pinch: Risk assessment and venom metering in the western black widow spider, *Latrodectus hesperus*. Anim. Behav..

[48] Wigger E., Kuhn-Nentwig L., Nentwig W. (2002). The venom optimisation hypothesis: A spider injects large venom quantities only into difficult prey types. Toxicon.

[49] Hayes W. (2008). The snake venom-metering controversy: Levels of analysis, assumptions, and evidence. The Biology of Rattlesnakes.

[50] Hayes W.K. (1995). Venom metering by juvenile prairie rattlesnakes, *Crotalus v. viridis*: Effects of prey size and experience. Anim. Behav..

[51] Hayes W.K., Herbert S.S., Rehling G., Gennaro J. (2002). Factors that influence venom expenditure in viperids and other snake species during predatory and defensive contexts. Biology of the Vipers.

[52] Young B.A., Lee C.E., Daley K.M. (2002). Do snakes meter venom?. BioScience.

[53] Dugon M.M., Arthur W. (2012). Prey orientation and the role of venom availability in the predatory behaviour of the centipede *Scolopendra subspinipes mutilans* (Arthropoda: Chilopoda). J. Insect Physiol..

[54] Edmunds M.C., Sibly R.M. (2010). Optimal sting use in the feeding behavior of the scorpion *Hadrurus spadix*. J. Arachnol..

[55] Lira A.F., Santos A.B., Silva N.A., Martins R.D. (2017). Threat level influences the use of venom in a scorpion species, *Tityus stigmurus* (Scorpiones, Buthidae). Acta Ethol..

[56] Nisani Z., Hayes W.K. (2011). Defensive stinging by *Parabuthus transvaalicus* scorpions: Risk assessment and venom metering. Anim. Behav..

[57] Cooper A.M., Nelsen D.R., Hayes W.K. (2015). The Strategic Use of Venom by Spiders. Toxinology: Evolution of Venomous Animal and Their Toxins.

[58] Hostettler S., Nentwig W. (2006). Olfactory information saves venom during prey-capture of the hunting spider *Cupiennius salei* (Araneae: Ctenidae). Funct. Ecol..

[59] Wullschleger B., Nentwig W. (2002). Influence of venom availability on a spider’s prey-choice behaviour. Funct. Ecol..

[60] Boevé J.-L., Kuhn-Nentwig L., Keller S., Nentwig W. (1995). Quantity and quality of venom released by a spider (*Cupiennius salei*, Ctenidae). Toxicon.

[61] Malli H., Imboden H., Kuhn-Nentwig L. (1998). Quantifying the venom dose of the spider *Cupiennius salei* using monoclonal antibodies. Toxicon.

[62] Malli H., Kuhn-Nentwig L., Imboden H., Nentwig W. (1999). Effects of size, motility and paralysation time of prey on the quantity of venom injected by the hunting spider *Cupiennius salei*. J. Exp. Biol..

[63] Schendel V., Rash L.D., Jenner R.A., Undheim E.A.B. (2019). The Diversity of Venom: The Importance of Behavior and Venom System Morphology in Understanding Its Ecology and Evolution. Toxins.

[64] Herzig V., King G.F., Undheim E.A. (2019). Can we resolve the taxonomic bias in spider venom research?. Toxicon X.

[65] Vassilevski A.A., Kozlov S.A., Grishin E.V. (2009). Molecular diversity of spider venom. Biochemistry.

[66] Morgenstern D., King G.F. (2013). The venom optimization hypothesis revisited. Toxicon.

[67] Dunbar J.P., Afoullouss S., Sulpice R., Dugon M.M. (2018). Envenomation by the noble false widow spider *Steatoda nobilis* (Thorell, 1875)–Five new cases of steatodism from Ireland and Great Britain. Clin. Toxicol..

[68] Atakuziev B.U., Wright C.E., Graudins A., Nicholson G.M., Winkel K.D. (2014). Efficacy of Australian red-back spider (*Latrodectus hasselti*) antivenom in the treatment of clinical envenomation by the cupboard spider *Steatoda capensis* (*Theridiidae*). Toxicon.

[69] Nentwig W., Friedel T., Manhart C. (1992). Comparative investigations on the effect of the venoms of 18 spider species onto the cockroach *Blatta orientalis* (*Blattodea*). Zool. Jb. Physiol..

[70] Betz O., Kölsch G. (2004). The role of adhesion in prey capture and predator defence in arthropods. Arthropod Struct. Dev..

[71] Blackledge T., Coddington J., Gillespie R. (2003). Are three-dimensional spider webs defensive adaptations?. Ecol. Lett..

[72] Forster L. (1995). The behavioural ecology of *Latrodectus hasselti* (Thorell), the Australian redback spider (*Araneae: Theridiidae*): A review. Rec. West. Aust. Mus..

[73] Anotaux M., Toscani C., Leborgne R., Châline N., Pasquet A. (2014). Aging and foraging efficiency in an orb-web spider. J. Ethol..

[74] Jackson R.R., Brassington R.J. (1987). The biology of *Pholcus phalangioides* (*Araneae, Pholcidae*): Predatory versatility, araneophagy and aggressive mimicry. J. Zool..

[75] Roberts M.J. (1995). Collins Field Guide: Spiders of Britain & Northern Europe.

[76] Herzig V., Ward R.J., dos Santos W.F. (2002). Intersexual variations in the venom of the Brazilian ′armed′ spider *Phoneutria nigriventer* (Keyserling, 1891). Toxicon.

[77] Vapenik Z., Nentwig W. (2000). The influence of hunger and breeding temperature on the venom production of the spider *Cupiennius salei* (Araneae, Ctenidae). Toxicon.

[78] Binford G.J. (2001). An analysis of geographic and intersexual chemical variation in venoms of the spider *Tegenaria agrestis* (Agelenidae). Toxicon.

[79] Zobel-Thropp P.A., Mullins J., Kristensen C., Kronmiller B.A., David C.L., Breci L.A., Binford G.J. (2019). Not so dangerous after all? Venom composition and potency of the pholcid (daddy long-leg) spider *Physocyclus mexicanus*. Front. Ecol. Evol..

[80] Team R.C. (2021). R: A Language and Environment for Statistical Computing.

[81] Burnham K.P., Anderson D.R. (2002). A practical information-theoretic approach. Model Selection and Multimodel Inference.

[82] Venables B., Ripley B.D. (2002). Modern Applied Statistics with S.

