# Supplementary material for: Worldwide Web: High Venom Potency and Ability to Optimize Venom Usage Make the Globally Invasive Noble False Widow Spider Steatoda nobilis (Thorell, 1875) (Theridiidae) Highly Competitive against Native European Spiders Sharing the Same Habitats
Source: Toxins (Basel). 2022 Aug 26;14(9):587. doi: 10.3390/toxins14090587 (PMC9500793; doi:10.3390/toxins14090587)
Supplement: Supplementary file 1 [file toxins-14-00587-s001.zip › toxins-1790641-supplementary.pdf]

# Supplementary Materials

**Table S1.** Details of the collection of specimens for each species of spiders used in the experiments: *A. similis*, *E. atrica*, *S. nobilis*, *P. phalangioides*, and *Z. x-notata*, including numbers of specimens sampled each season and details of each sampling location. PBIVA = Predatory Behaviour Influenced by Venom Availability.

| Experiment | Spider species | Total number of specimens per experiment | Year | Season | Location |
|---|---|---|---|---|---|
| PBIVA | *S. nobilis* | 120 | 2019 | Autumn (*n* = 120) | Dublin (N=120) |
| Venom yield v size | *S. nobilis* | 550 | 2018–2021 | Spring (*n* = 88)<br>Summer (*n* = 180)<br>Autumn (*n* = 282) | Dublin, Galway, Longford |
| Venom yield v size | *E. atrica* | 139 | 2018–2021 | Summer (*n* = 114)<br>Autumn (*n* = 25) | Dublin, Galway, Longford |
| Venom yield v size | *A. similis* | 265 | 2018–2021 | Summer (*n* = 83)<br>Autumn (*n* = 182) | Dublin, Galway, Longford |
| Toxicity assays | *S. nobilis* | 120 | 2019–2021 | Autumn (*n* = 120) | Dublin, Galway |
| Toxicity assays | *E. atrica* | 105 | 2019–2021 | Autumn (*n* = 105) | Dublin, Galway, Longford |
| Toxicity assays | *A. similis* | 135 | 2019–2021 | Autumn (*n* = 135) | Dublin, Galway, Longford |
| Toxicity assays (prey) | *Z. x-notata* | 195 | 2019–2021 | Autumn (*n* = 195) | Galway |
| Predation observations | *S. nobilis* | 40 | 2019 | Autumn (40) | Dublin (*n* = 40) |
| Predation observations | *E. atrica* | 10 | 2019 | Autumn (10) | Galway (*n* = 10) |
| Predation observations | *A. similis* | 10 | 2019 | Autumn (10) | Galway (*n* = 10) |
| Predation observations | *Z. x-notata* | 10 | 2019 | Autumn (10) | Galway (*n* = 10) |
| Predation observations | *P. phalangioides* | 10 | 2019 | Autumn (10) | Galway (*n* = 10) |

**Total per season**

*P. Phalangioides:* Autumn 2019 (*n* = 10)

*Z. x-notata:* Autumn 2019–2021 (*n* = 205)

*A. similis:* Autumn 2018–2021 (*n* = 327); Summer 2018–2021 (*n* = 83)

*E. atrica*: Autumn 2018–2021 (140); Summer 2018–2021 (*n* = 114)

*S. nobilis*: Spring 2018–2021 (*n* = 88); Summer 2018–2021 (*n* = 180); Autumn 2018–2021 (*n* = 562)
